# Postextraction Alveolar Preservation and Use of the Crown of the Extracted Tooth as a Temporary Restoration

**DOI:** 10.1155/2019/4262067

**Published:** 2019-03-03

**Authors:** Andrea Rodríguez Moreno, Mariné Ortiz Magdaleno, Marissa Muñoz Islas, Jorge Armando Villar Mercado, María del Pilar Goldaracena Azuara, Everardo Ruiz Cruz, Gabriel Fernando Romo Ramírez

**Affiliations:** ^1^Autonomous University of San Luis Potosí, Specialty in Aesthetic, Cosmetic, Restorative, and Implantological Dentistry, Faculty of Stomatology, Av. Dr. Manuel Nava #2, Zona Universitaria, 78290 San Luis Potosí, Mexico; ^2^Autonomous University of San Luis Potosí, Specialty in Periodontics, Faculty of Stomatology, Av. Dr. Manuel Nava #2, Zona Universitaria, 78290 San Luis Potosí, Mexico

## Abstract

Ranging from the extraction of teeth from anterior sector commitment to the loss of bone and soft tissues, alveolar preservation is a regenerative technique that employs biomaterials that induce the preservation of bone tissue and minimize resorption. Placement of provisional restorations at the defect site maintains the stability of the soft tissues and aesthetics at which the definitive restoration is found. The objective of this clinical case was alveolar preservation postextraction of the anterior tooth that presented a coronal fracture, placing the clinical crown of the extracted tooth as the provisional restoration to maintain stability and to avoid soft tissue collapse and compromise to an aesthetic area.

## 1. Introduction

Losing a tooth involves surgical, periodontal, restorative, and aesthetic procedures. The surgical procedure for the extraction of a tooth with a poor prognosis, due either to caries, periodontal conditions, or trauma in the anterior sector, causes a series of dimensional and morphological changes in the soft and hard tissues [1]. The loss of bone volume is a factor that affects the placement of implants: if placement of an implant at the extraction site is desired, then ideally, at the time of surgery, tissue regeneration should be performed by placement of grafts or biomaterials with osteoinductive properties and use of osteogenics, which regenerate the lost tissue for later implant placement [2]. When a tooth is extracted, a process of bone resorption usually occurs, causing atrophy of the alveolar ridge. To prevent this type of defect, therapeutic treatments have emerged that preserve the alveolar tissue and maintain the soft tissue and the emerging dimensions of the alveolar contours [3–5].

After dental surgery, the soft tissue collapses, and the bone tissue that makes up the alveolus undergoes biological healing processes that affect its dimensions both in volume and in morphology. In addition, it is not possible to predict the morphology of the defect site because the width of the reabsorbed bone (vestibular-palatal) is increased; it is related to the thickness of the alveolar external crest of the maxilla in vertical terms (apico-coronal) [6, 7]. Techniques of alveolar preservation are the set of techniques aimed at the preservation of the ridge volume determined at the time of tooth extraction, minimizing reabsorption of the alveolar ridge [8, 9]. This is intended to decrease; however, it does not completely eliminate its resorption horizontally, since its objective is to maintain bone volume [10]. The alveolar ridge has been reported to lose 50% of its height and width during the first 6 months; therefore, studies have reported that socket-sealing surgery minimizes the loss of alveolar bone and soft tissues [11, 12].

Alveolar preservation is indicated when, for surgical reasons, there is a contraindication to placing a postextraction implant, when the desire is to improve and restore the loss of bone tissue and soft tissue that have been lost by a traumatic dental extraction and the presence of dehiscence and fenestration [13–15]. The bone morphology of the maxilla in the anterior sector compromises the aesthetics of the soft tissues; thus, planning the definitive restoration will depend on the design of the provisional restoration. Once alveolar preservation has been carried out, provisionalization of the edentulous sector should be considered with the use of a provisional restoration that does not affect the regeneration of bone and soft tissues. An accurate diagnosis and appropriate management of postextraction alveolar preservation techniques allow us to succeed in the predictability of the clinical case.

This case report will show a clinical case in which a fracture of coronal tooth #21 was diagnosed without the possibility of prosthetic rehabilitation. Dental extraction and alveolar preservation were indicated, while placement of the postextraction implant was contraindicated due to the lack of sufficient thickness in the buccal vestibular crest, and the provisional restoration consisted of the same fractured autologous clinical crown of tooth #21. This case report presents a step-by-step alveolar preservation protocol and provisional restoration.

## 2. Case Description

A 41-year-old female patient attended the Specialty in Cosmetic Dentistry, Cosmetic, Restorative, and Implantology Clinic of the Faculty of Stomatology, Autonomous University of San Luis Potosí, Mexico, for consultation due to the mobility of the clinical crown of tooth #21 because of a trauma The patient did not have relevant medical records, and thus, we requested a computerized axial tomography (CT) scan to assess the thickness of the buccal bone table and the coronal fracture.

### 2.1. Clinical Examination

**Figure 1:**
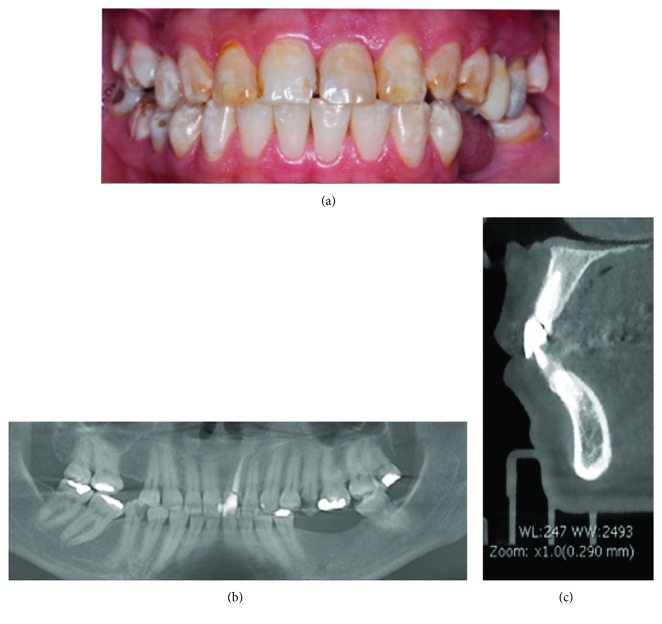
Anterosuperior sector: the clinical crown fracture of tooth #21 is clinically not observed (a); panoramic x-ray (b) and computerized axial tomography (c).

In the intraoral clinical evaluation (Figure 1(a)), mobility grade 3 of the clinical crown of tooth #21 was diagnosed. A panoramic X-ray was taken to assess the root morphology of tooth #21 (Figure 1(b)). The CT revealed that tooth #21 was fractured at the cervical level of the clinical crown (Figure 1(c)) and that dental extraction was indicated without the possibility of placing a postextraction implant, since there was insufficient thickness of the external vestibular table and the available remnants would lead to a fenestration or lack of primary stability.

### 2.2. Treatment Plan

Atraumatic extraction of tooth #21 was planned, along with alveolar preservation to minimize bone reabsorption, placing a provisional restoration with the same autologous clinical crown of extracted tooth #21, which would maintain the same gingival level as that of the natural tooth.

### 2.3. Dental Extraction and Alveolar Preservation

**Figure 2:**
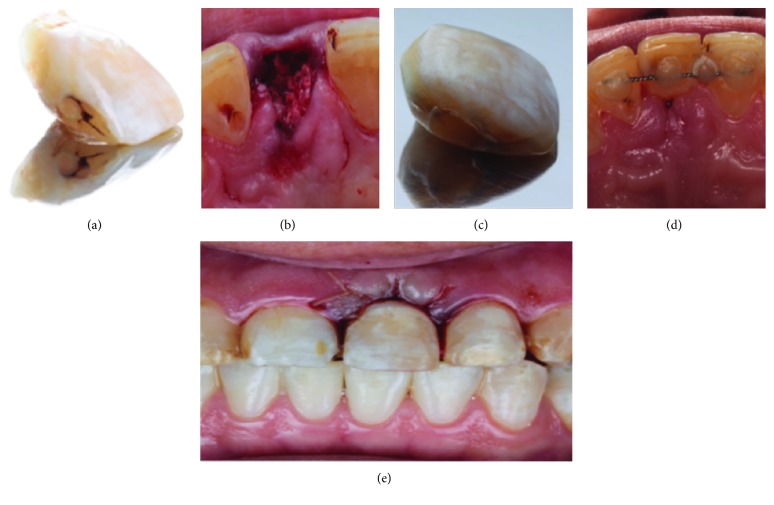
Extracted autologous clinical crown (a); filling of the alveolus with InterOss® (b); formation of the ovoid pontic in the cervical part of the clinical crown (c); adherence with conventional composite and orthodontic wire in the palatal sides of teeth #11 and #22 (d); the provisional restoration is left out of occlusion in intercuspid position (e).

The tooth extraction procedure for tooth #21 consisted of first separating the fractured clinical crown (Figure 2(a)) to subsequently and atraumatically extracting the root remnant with #65 forceps. The alveolus was curetted to eliminate any remaining granulation tissue and periodontal ligament, and to induce bleeding, an InterOss® alveolus was placed in the 0.5 g bone xenograft (Anorganic Cancellous Bone Graft Granules) (Figure 2(b)) to subsequently seal the alveolus surface with a collagen membrane of 8 mm × 12 mm (Colla Tape, Zimmer) sutured with Vicryl 4-0.

### 2.4. Provisionalization

Once the clinical crown was separated from the root of tooth #21, it was washed with physiological serum and disinfected with 2% chlorhexidine, and a pulpotomy of the crown was performed to eliminate the pulp tissue and to seal the pulp chamber with a conventional composite and the ovoid pontic (Figure 2(c)). The crown was fused with a metal ligature and conventional composite on the palatal surfaces of teeth #11 and #22 (Figure 2(d)). The cervical part of the provisional crown should exert slight pressure in the area of the extraction, thus beginning to create a natural emergence profile for at least 3 months after this alveolar preservation to set the implant in place. The provisional restoration was omitted from the occlusion in the opening-closing and mandibular eccentric movements (Figure 2(e)).

### 2.5. Postoperative Follow-Up

**Figure 3:**
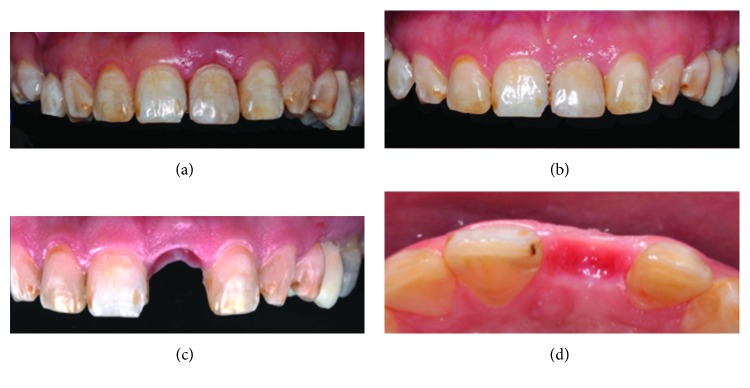
The patient is scheduled for a weekly appointment for the removal of the sutures (a); good aesthetic after 1 month with papilla preservation (b); the provisional restoration is removed (c–d).

**Figure 4:**
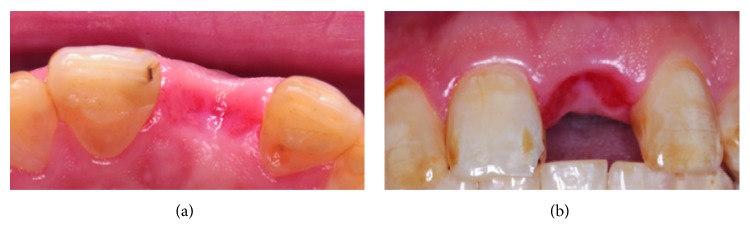
Final result after 3 months with the autologous clinical crown as a provisional restoration (a); emerging dimensions of the alveolar contours (b).

**Figure 5:**
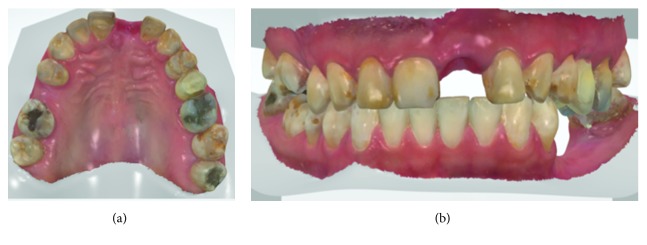
Images obtained from an intraoral scanner to plan the placement of the implant.

The patient was scheduled for an appointment 1 week after the tooth extraction to remove the sutures (Figure 3(a)) and to evaluate possible inflammation after extraction. Nevertheless, a healthy gum was observed, including a coral pink color, and the dental papilla had maintained its dimensions. At 1 month after surgery, the soft tissue was evaluated and was not collapsed, did not present gingival inflammation, and demonstrated a maintained confirmation of the volume and dimension of the papillae (Figure 3(b)). The autologous clinical crown was removed to evaluate the edentulous flange. Next, we evaluated the conformation of the ovoid pontic, and we decided to add a small amount of the conventional composite on the pontic to provide a natural emergence profile (Figures 3(c) and 3(d)). At 3 months after the extraction, the level of the soft tissue was observed (Figures 4(a) and 4(b)). An intraoral scanner (3Shape) was applied for dental implant placement and surgical guidance (Figures 5(a) and 5(b)).

## 3. Discussion

The objective of this clinical case was to maintain the biological parameters of bone and soft tissues by performing postextraction alveolar preservation and using the complete crown of the extracted tooth as a provisional restoration; the results revealed success in the contouring and festooning of the gum without a loss of gingival papillae, as well as preservation of the integrity and architecture of the soft tissues. Advantages obtained in this manner of provisionalization reduce the probability of soft tissue collapse, and alveolar preservation after extraction limits the loss of bone tissue. Further scope of this research was to disseminate information concerning the importance of an adequate way to provisionalize edentulous spaces in an aesthetically and functionally compromised area such as the anterior superior sector of the maxilla and the preservation of an alveolus postextraction that minimizes bone resorption to the greatest extent possible.

The loss of a tooth in the anterior maxilla gives rise to a series of problems that, if not resolved at the correct time, can be detrimental to the implant and restorative features. However, loss of a tooth from an aesthetic zone also has an impact on the patient, both in his or her manner of speaking and masticatory function, and it can even affect socialization due to sensing the absence of the tooth [16].

In this clinical case, an adequate diagnosis and treatment plan was shown to successfully predict the definitive restoration by the placement of an adequate provisional restoration, due to the fracture of the clinical crown of tooth #21. Alveolar preservation postextraction was proposed to maintain the morphology and dimensions of the alveolus and to decrease the process of bone resorption after the dental extraction, as well as the time required for bone healing, in this manner avoiding the collapse of soft tissues.

Studies have shown that the loss of alveolar ridge volume postextraction consists of an irreversible process that forms part of the biological processes and alveolar bone changes due to the loss of a tooth, involving horizontal and vertical reductions [17, 18]. The effects of filling with bone-grafting materials and of not filling with any material have revealed variable dimensional changes, and a superior impact of alveolar preservation can be clearly observed [19]. However, resorption after tooth extraction may be limited, but it cannot be eliminated with alveolar preservation, since the formation of new bone is not always promoted at the histological level [8].

*In vivo* and *in vitro* studies have revealed the study of a wide variety of materials as substitutes for the regeneration of bone tissue, including autografts, allografts, xenografts, and alloplasts, maintaining the bone volume after extraction, compensating for the degree of bone resorption, and accelerating the formation of bone tissue [20, 21]. In this clinical case, we decided to use a natural hydroxyapatite bone-grafting material derived from bovine bone, which acts as a guide for body fluids, growth factors, and cells. In contrast, the wound dressing consisted of a resorbable regenerative membrane, which is indicated for inducing hemostasis and coating of surgical beds [22]. Prior to filling of the alveolus with the regenerative material in this clinical case, a collagen membrane was placed; this membrane functions as a dressing that controls bleeding by stabilizing the blood clot and accelerating the healing process [23]. These membranes have been shown to be effective at protecting the alveolus and graft material from the oral environment through a barrier effect, preventing the rapid growth of soft tissues [24].

The placement of a provisional restoration should be scheduled as soon as possible after the final restoration, regarding the shape and dimensions of the tooth, as well as the emergence profile and axial inclination following positioning of the root of the missing tooth. At the level of the soft tissues after dental extraction, the entrance to the alveolus is empty, and healing occurs by secondary intention, such that subsequent cell proliferation causes an increase in the volume in the outer part of the alveolus, and the behavior of the soft tissues depends on the dimensional changes in the bone tissue [17].

An advantage of this clinical case was that the margin of the fracture occurred at the cervical level of the tooth without compromising the clinical crown. Therefore, it could be utilized as a provisional restoration. The postoperative images at 1 week and 1 month demonstrated no collapse of the soft tissues or loss of the volume and dimensions of the papillae, since the crown maintained the contact points.

Alveolar preservation is a key factor for minimizing bone loss; however, the fabrication of a suitable provisional restoration will help to maintain soft tissue integrity without necessarily being interrelated. The options for provisionalizing unitary edentulous spaces postextraction with alveolar preservation include fixed partial prostheses in the case of multiple restorations or a Maryland-type restoration, which is an adhesive prosthesis. Importantly, the provisional restoration should not exert a strong pressure at the defect site to avoid compromising the vascularization and healing processes. After it has healed, it is recommended to remove the provisional restoration, add a conventional composite for the formation of the ovoid pontic, and configure the crest profile in the desired manner [20].

## 4. Conclusion

In this case report, we showed a successful outcome in terms of the integrity, margin, and architecture of the soft tissues after extraction of a superior central tooth, demonstrating that placement of the patient's autologous clinical crown as a provisional crown provides stability to the soft tissues and offers the creation and modeling of an emergence profile as natural as the extracted tooth itself. It also offers aesthetics that provide the patient with assurance to speak and smile without the need for placement of a temporary removable restoration.
